# Supplementary figures and images for: QTL mapping and candidate gene analysis of low temperature germination in rice (Oryza sativa L.) using a genome wide association study
Source: PeerJ. 2022 May 11;10:e13407. doi: 10.7717/peerj.13407 (PMC9107303; doi:10.7717/peerj.13407)

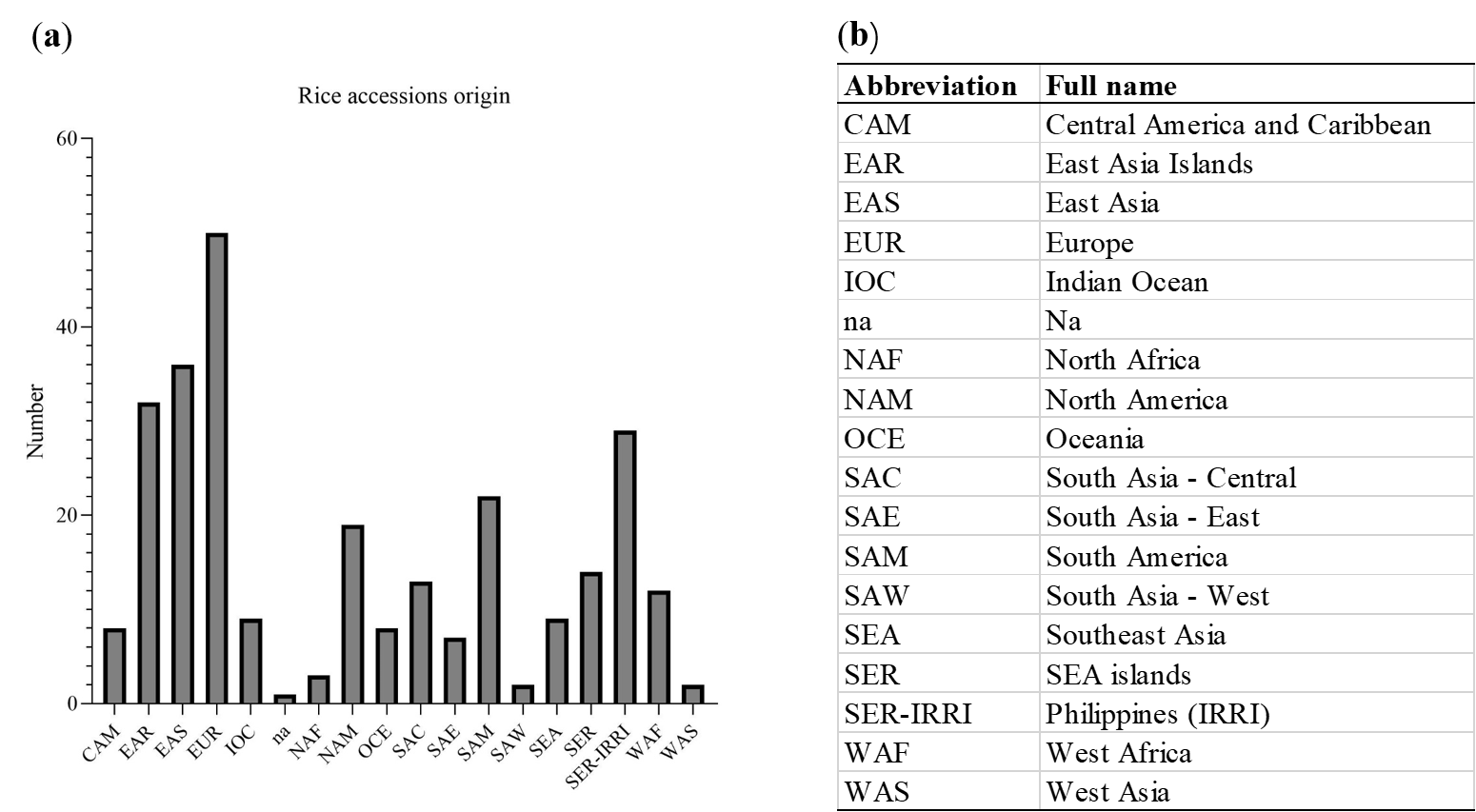

Supplement: Supplemental Information 1 — (a) The distribution of accessions from different regions worldwide. (b) Specific information for abbreviations of regions [file peerj-10-13407-s001.png]

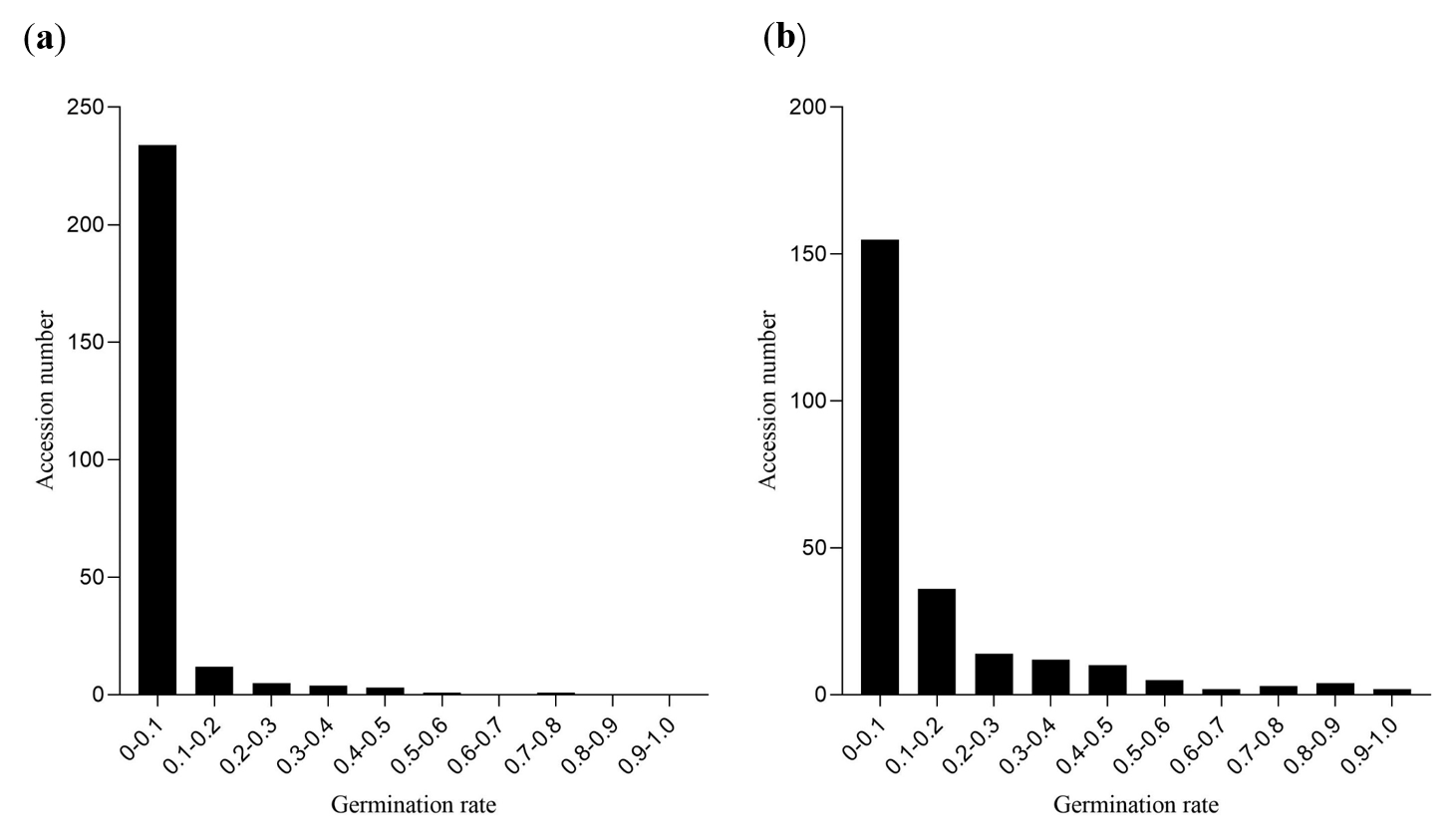

Supplement: Supplemental Information 2 — (a) The distribution of germination rate on D6 of 2019. (b) The distribution of germination rate on D6 of 2020. [file peerj-10-13407-s002.png]

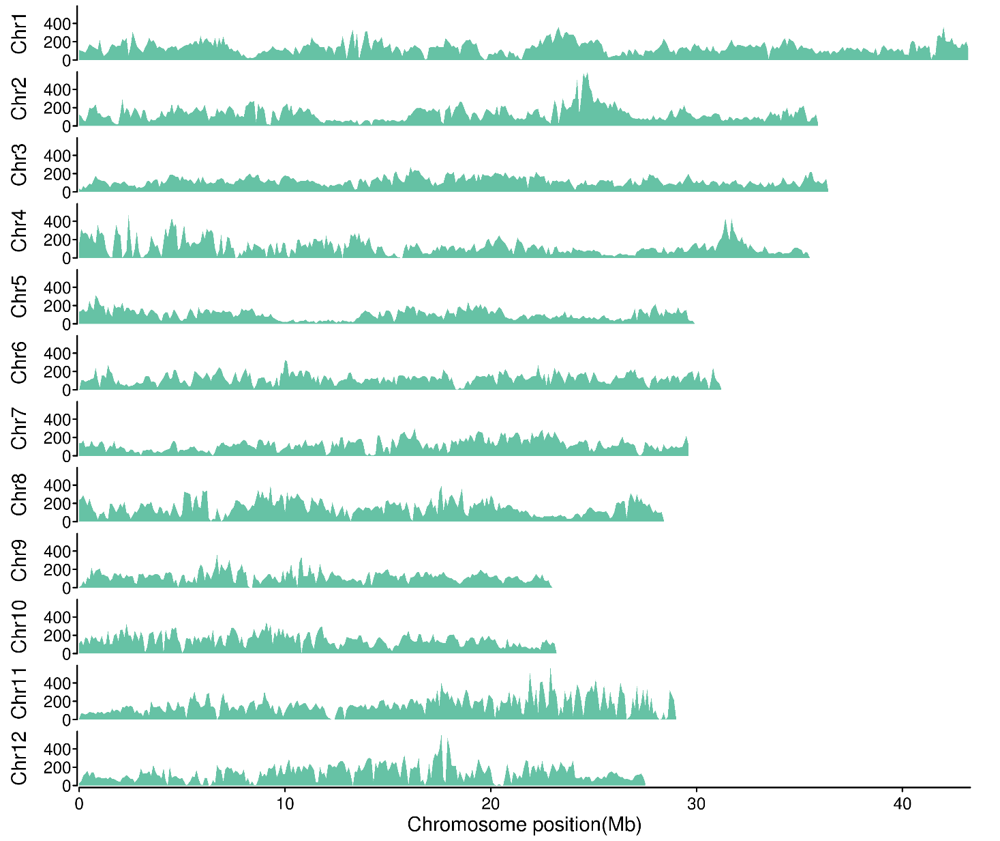

Supplement: Supplemental Information 3 [file peerj-10-13407-s003.png]

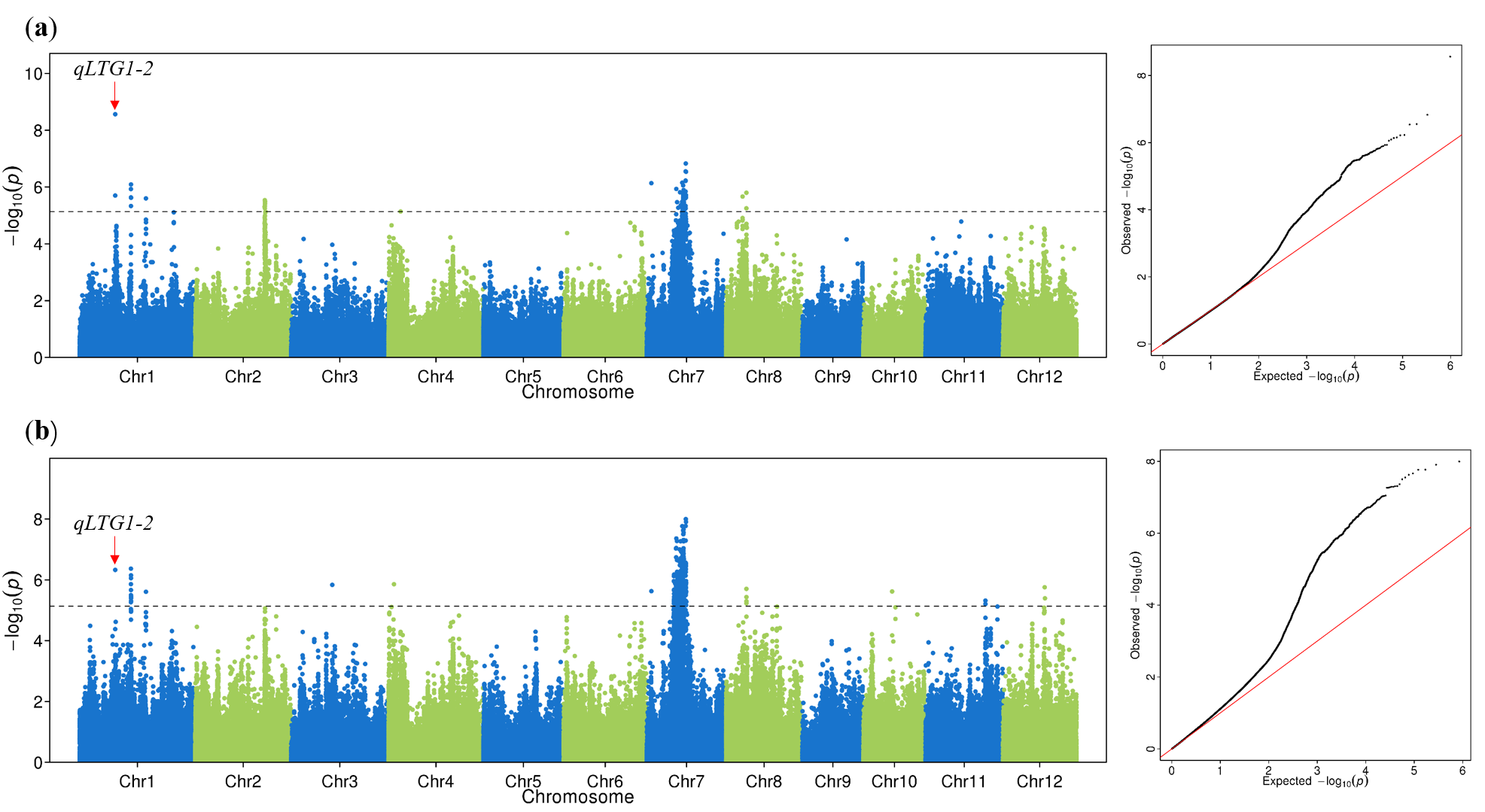

Supplement: Supplemental Information 4 — (a) A GWAS performed on 2020D6 using FaST-LMM. (b) A GWAS performed on 2020D6 using GEMMA. The red arrow represents the repeatedly identified qLTG1-2 in the study. The dashed horizontal line represents the suggestive threshold (P = 7.34 × 10^−6^, −log10P = 5.13). [file peerj-10-13407-s004.png]

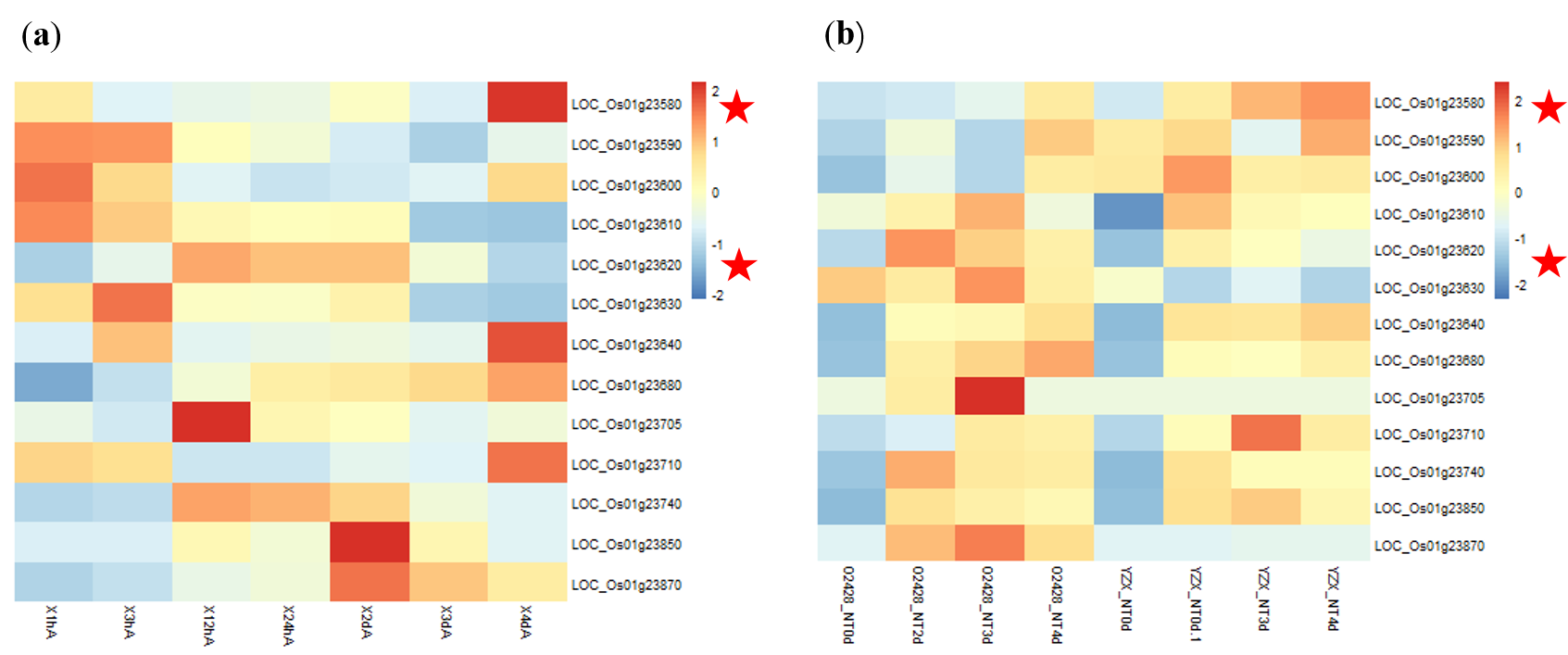

Supplement: Supplemental Information 5 — (a) Transcriptomic data from Narsai et al. (2017). (b) Transcriptomic dataset from Yang et al. (2020). [file peerj-10-13407-s005.png]

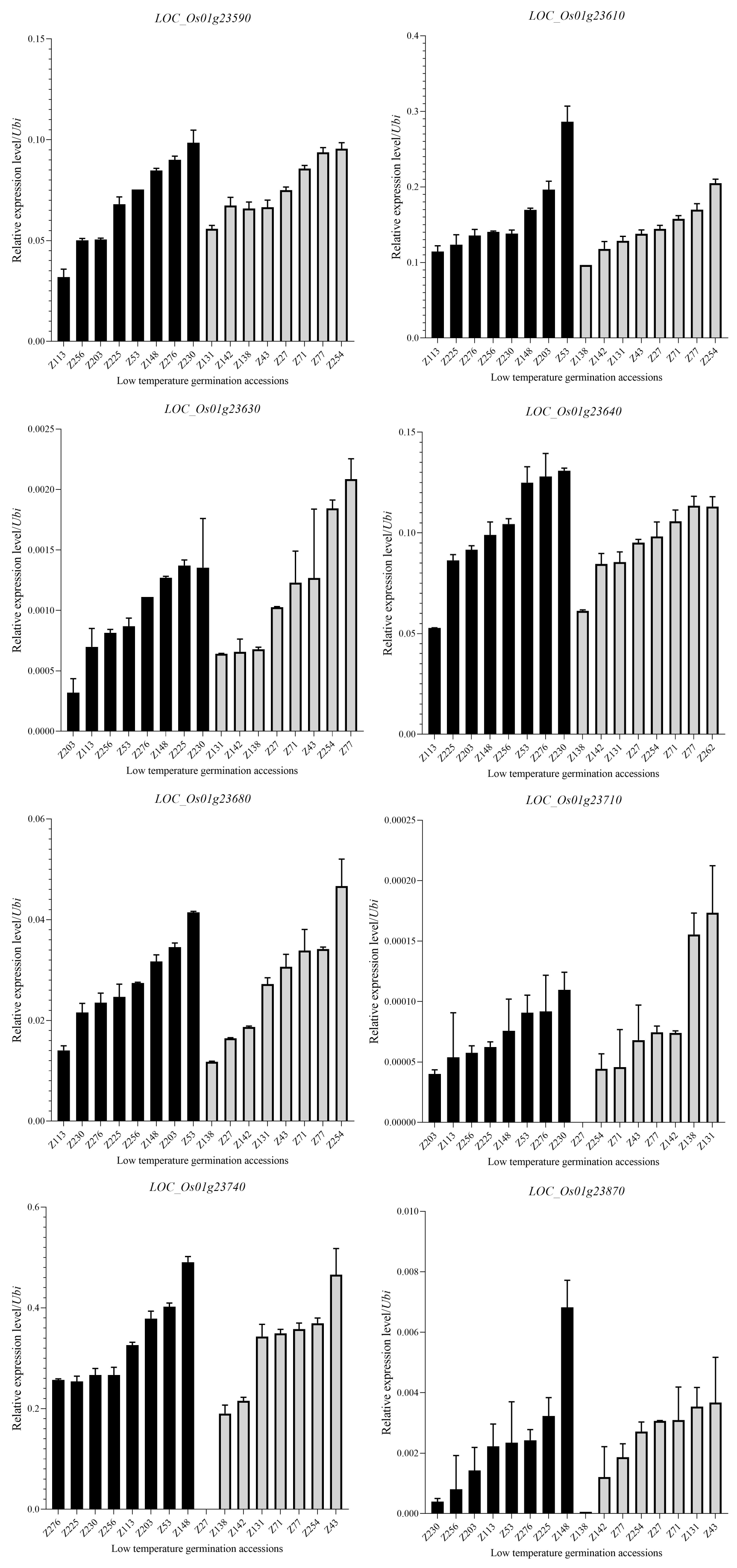

Supplement: Supplemental Information 6 [file peerj-10-13407-s006.png]

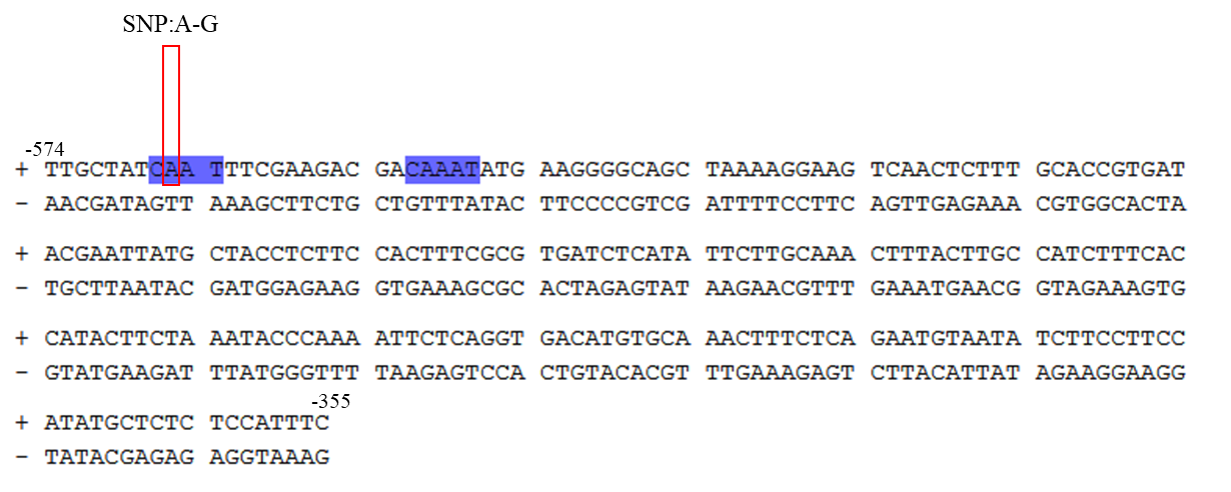

Supplement: Supplemental Information 7 — Blue block represents CAAT-box; red box represents Chr.1_13285882 which located within 1 kb region ahead of the CDS region of OsSar1a. [file peerj-10-13407-s007.png]
